# Hepatitis E Virus Seroprevalence among Adults, Germany

**DOI:** 10.3201/eid1810.111756

**Published:** 2012-10

**Authors:** Mirko S. Faber, Jürgen J. Wenzel, Wolfgang Jilg, Michael Thamm, Michael Höhle, Klaus Stark

**Affiliations:** Robert Koch Institute, Berlin, Germany (M.S. Faber, M. Thamm, M. Höhle, K. Stark);; and University of Regensburg, Regensburg, Germany (J.J. Wenzel, W. Jilg)

**Keywords:** Hepatitis E virus, cross-sectional study, HEV, hepatitis E, seroprevalence, Germany, viruses, hepatitis, adults

## Abstract

We assessed hepatitis E virus (HEV) antibody seroprevalence in a sample of the adult population in Germany. Overall HEV IgG prevalence was 16.8% (95% CI 15.6%–17.9%) and increased with age, leveling off at >60 years of age. HEV is endemic in Germany, and the lifetime risk for exposure is high.

In industrialized countries, hepatitis E virus (HEV) has long been regarded as a rare imported infection. However, sporadic cases without travel to disease-endemic areas and caused by genotype 3 are being increasingly reported (*1*,*2*). Epidemiologic and molecular studies have implicated undercooked pork and wild boar products as a source of HEV infection (*3*–*5*). An unexpectedly high prevalence of HEV-specific antibodies, e.g., among blood donors, has been shown by several studies in Europe and the United States (*6*–*11*).

In Germany, the number of notified hepatitis E cases rose from <50 annually in 2001–2003 to 238 in 2011 (incidence 0.3/100,000 population); the proportion of autochthonous cases increased from 30%–40% to 78%. We conducted a study to determine HEV seroprevalence in Germany’s adult population and associations with sociodemographic characteristics by using an assay highly sensitive for HEV genotype 3.

## The Study

We assessed HEV seroprevalence in a large subsample (n = 4,422) of the 2008–2011 German Health Examination Survey for Adults (Deutscher Erwachsenen Gesundheitssurvey; www.degs-studie.de), a 2-stage national probability sample that assessed the health status in the general population. The sampling frame comprised persons 18–79 years of age whose principal residence was in Germany and who were fluent in German. Overall response was 48.4% (7,116 respondents). Our subsample reflects the total adult population with respect to age, sex, and geographic region, but persons with migration background are underrepresented (non-German citizenship 4.6% in the sample vs. 8.7% in the total adult population).

Serum samples were screened for HEV IgG by using the *recom*Line HEV-IgG/IgM immunoassay (Mikrogen, Neuried, Germany). The assay is based on 7 recombinantly expressed antigens of genotypes 1 and 3 of open reading frames 2 and 3. According to the manufacturer’s and our data (J.J. Wenzel et al., unpub. data), the test is 97%–100% sensitive for detecting acute or previous HEV infections. Test strips were scanned with the semiautomatic *recom*Scan software (Mikrogen). The intensity of 3 quality assurance and other bands was determined by densitometrical detection algorithms. Each antigen band with an intensity greater or equal to the cutoff was assigned a point value. The final results were classified into 3 categories: no antibodies detectable (negative), test inconclusive (borderline), and antibodies detectable (positive). Persons whose test results were borderline (n = 70) were excluded from further analysis.

We poststratified the remaining survey population (n = 4,352) by age group and location of residence (16 states) to account for per protocol oversampling in eastern Germany and to restore the distribution of age groups to match the distribution in the total population. Weighted seroprevalence estimates were calculated by using survey-weighted logistic regression. Associations between demographic characteristics and seropositivity were analyzed by using adjusted Wald test p values. We also estimated mean annual incidence of HEV seroconversion from the seroprevalence data by using a catalytic model with age-constant force of infection, similar to that of Faramawi et al. (*12*). Detailed methods and underlying assumptions are described in the Technical Appendix.

The 4,352 persons who were included in the analysis were from 108 communities of all federal states in Germany (Table 1). Weighted prevalence of HEV IgG was 16.8% (95% CI 15.6%–17.9%); prevalence ranged from 6.1% (95% CI 4.5%–7.8%) in the 18–34-year age group to >20% in the >50-year groups, with a maximum of 26.4% (95% CI 21.6%–31.1%) in the 60–64-year group (Figure). In the univariable analysis (Table 2), only age group was significantly associated with seropositivity (p<0.01); results were not significant for sex (p = 0.97), residence (northern/middle/southern Germany, p = 0.29; west/east, p = 0.43), or population of municipality (4 categories; p = 0.10). In separate multivariable models, each including age group and 1 other variable, age remained the only significant variable. Mean annual incidence of HEV seroconversion estimated from the catalytic model was 3.9 (95% CI 3.6%–4.2%) per 1,000 population.

**Table 1:** Comparison of demographic characteristics of persons in study of hepatitis E virus seroprevalence and general adult population, Germany, 2008–2011

Characteristic	% Study population, n = 4,352	% Total population, n = 64,139,871*
Age group, y		
18–19	3.2	2.9
20–29	12.3	15.5
30–39	11.5	15.6
40–49	18.4	21.7
50–59	19.5	17.9
60–69	19.7	14.3
70–79	15.4	12.2
Female sex	51.3	50.3
State of residence		
Baden-Württemberg	11.4	13.0
Bavaria	10.7	15.2
Berlin	3.4	4.4
Brandenburg	6.2	3.2
Bremen	0.9	0.8
Hamburg	1.4	2.2
Hesse	6.4	7.4
Mecklenburg-Vorpommern	3.8	2.1
Lower Saxony	11.2	9.5
North Rhine-Westphalia	14.8	21.6
Rhineland-Palatinate	5.1	4.8
Saarland	2.2	1.3
Saxony	7.1	5.3
Saxony-Anhalt	6.2	3.0
Schleswig-Holstein	2.6	3.4
Thuringia	6.7	2.9

*Total no. adults 18–79 years of age as of December 31, 2009 (www.destatis.de).

**Figure:**
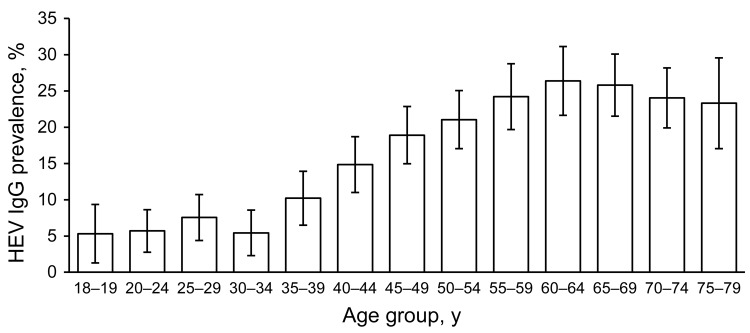
Estimated prevalence of hepatitis E virus (HEV) IgG, by age group, Germany, 2008–2011. Error bars indicate 95% CIs.

**Table 2:** Univariable survey-weighted logistic regression estimates of seroprevalence of hepatitis E virus IgG, by age, sex, and location of residence, Germany, 2008–2011*

Characteristic	Seroprevalence, % (95% CI)
Total	16.8 (15.6–17.9)
Age group, y	
18–19	5.3 (2.5–11.1)
20–29	6.6 (4.8–9.2)
30–39	8.0 (5.8–10.8)
40–49	16.9 (14.3–19.9)
50–59	22.6 (19.7–25.7)
60–69	26.1 (23.0–29.4)
70–79	23.8 (20.4–27.5)
Sex	
M	16.8 (15.2–18.5)
F	16.7 (15.2–18.5)
Place of residence (north–south)	
Northern states	15.5 (13.5–17.6)
Middle states	16.6 (14.8–18.6)
Southern states	17.9 (15.8–20.1)
Place of residence (east-west)	
Western states	16.6 (15.2–18.0)
Eastern states	17.6 (15.6–19.7)
Population of municipality	
<5,000	17.9 (15.3–20.8)
5,000–<20,000	17.5 (15.2–20.1)
20,000–<100,000	14.4 (12.4–16.6)
>100,000	17.7 (15.7–20.0)

*n = 4,352.

## Conclusions

We found an overall HEV seroprevalence of 16.8% among adults in Germany; seroprevalence increased with age but was not dependent on sex or location of residence. Similarly high seroprevalence was found in blood donors in Denmark (20.6%), southwestern England (16%), and the United States (18%) (*6*,*7*,*9*). Estimates from Switzerland and the Netherlands, on the other hand, are considerably lower (*10*,*11*). Reasons for these differences could be effects of sample selection, different lifetime exposures (e.g., to foods that may serve as transmission vehicles), or use of different test systems with varying sensitivity. Mansuy et al. recently reported 53% prevalence of HEV antibodies in blood donors in southwestern France (*13*), a figure considerably higher than the 17% prevalence reported earlier for the same geographic region, when a different test system was used (*8*). The assay applied in our study was designed to also detect previous infections with HEV genotype 3; therefore, it is likely to be more sensitive than assays used in previous studies.

Our data show that HEV exposure is common among the general adult population in Germany, which is consistent with increasing evidence for pigs as a reservoir for foodborne transmission of HEV in industrialized countries. HEV seroprevalence is high in domestic pig herds in Germany and other countries; closely related HEV strains were found in pig livers on retail sale and in autochthonous cases of hepatitis E, and HEV seroprevalence was higher in persons with occupational exposure to pigs than in control groups (*4*,*10*,*14*,*15*).

Our data and other studies (*11*–*14*) have shown no significant difference in seroprevalence between sexes, despite an assumed higher frequency of alimentary or occupational exposure and the higher incidence of clinical cases among men. This finding may indicate sex-specific differences in disease development or application of laboratory testing or that foods frequently consumed by both sexes play a substantial role as vehicles for transmission. These results also may highlight the lack of evidence for 1 main risk factor or food vehicle (*14*).

The strong association between age and HEV seroprevalence in our study most likely reflects cumulative lifetime exposure to the virus. However, HEV seroprevalence remains relatively stable from 18 to 34 years of age, which could indicate a birth cohort effect resulting from a decrease in the overall risk over the past few decades, as was reported for Denmark (*6*). The leveling off of the seroprevalence above age 60 years could be caused by a loss of antibodies in the elderly.

We found a striking difference between the estimated annual incidence of seroconversion and the relatively low incidence of notified disease in Germany. Besides underdiagnosis, a possible explanation is a high proportion of asymptomatic infections.

The survey response rate is similar to those achieved in other comprehensive national health examination surveys in Europe. In the statistical analysis, we corrected for differences in the distribution of age and place of residence between the sample and the general population. However, foreign-born persons are underrepresented in the survey because participation required fluency in German. A high proportion of foreign-born persons in Germany are Muslim, and their avoidance of raw pork products likely decreases their risk for HEV infection, which means we may have slightly overestimated the seroprevalence in the adult general population.

Autochthonous HEV infections are common in the industrialized world, and testing for HEV is indicated in patients with acute hepatitis, irrespective of history of travel to developing countries. Recommendations against eating undercooked pork products exist, but further studies are needed to specify implicated food products and to better target recommendations for safer food production, preparation, and consumption.

Technical Appendix Details of mathematical methods used to compute the yearly incidence from the seroprevalence data in the manuscript.

## References

[1] Mushahwar IK. Hepatitis E virus: molecular virology, clinical features, diagnosis, transmission, epidemiology, and prevention. J Med Virol. 2008;80:646–58. 10.1002/jmv.21116 18297720

[2] Dalton HR, Hazeldine S, Banks M, Ijaz S, Bendall R. Locally acquired hepatitis E in chronic liver disease. Lancet. 2007;369:1260. 10.1016/S0140-6736(07)60595-9 17434400

[3] Adlhoch C, Wolf A, Meisel H, Kaiser M, Ellerbrok H, Pauli G. High HEV presence in four different wild boar populations in East and West Germany. Vet Microbiol. 2009;139:270–8. 10.1016/j.vetmic.2009.06.032 19595519

[4] Wenzel JJ, Preiss J, Schemmerer M, Huber B, Plentz A, Jilg W. Detection of hepatitis E virus (HEV) from porcine livers in southeastern Germany and high sequence homology to human HEV isolates. J Clin Virol. 2011;52:50–4. 10.1016/j.jcv.2011.06.006 21742549

[5] Wichmann O, Schimanski S, Koch J, Kohler M, Rothe C, Plentz A, Phylogenetic and case-control study on hepatitis E virus infection in Germany. J Infect Dis. 2008;198:1732–41. 10.1086/593211 18983248

[6] Christensen PB, Engle RE, Hjort C, Homburg KM, Vach W, Georgsen J, Time trend of the prevalence of hepatitis E antibodies among farmers and blood donors: a potential zoonosis in Denmark. Clin Infect Dis. 2008;47:1026–31. 10.1086/591970 18781880 PMC2803052

[7] Dalton HR, Stableforth W, Thurairajah P, Hazeldine S, Remnarace R, Usama W, Autochthonous hepatitis E in Southwest England: natural history, complications and seasonal variation, and hepatitis E virus IgG seroprevalence in blood donors, the elderly and patients with chronic liver disease. Eur J Gastroenterol Hepatol. 2008;20:784–90. 10.1097/MEG.0b013e3282f5195a 18617784

[8] Mansuy JM, Legrand-Abravanel F, Calot JP, Peron JM, Alric L, Agudo S, High prevalence of anti–hepatitis E virus antibodies in blood donors from south west France. J Med Virol. 2008;80:289–93. 10.1002/jmv.21056 18098159

[9] Meng XJ, Wiseman B, Elvinger F, Guenette DK, Toth TE, Engle RE, Prevalence of antibodies to hepatitis E virus in veterinarians working with swine and in normal blood donors in the United States and other countries. J Clin Microbiol. 2002;40:117–22. 10.1128/JCM.40.1.117-122.2002 11773103 PMC120098

[10] Bouwknegt M, Engel B, Herremans MM, Widdowson MA, Worm HC, Koopmans MP, Bayesian estimation of hepatitis E virus seroprevalence for populations with different exposure levels to swine in the Netherlands. Epidemiol Infect. 2008;136:567–76. 10.1017/S0950268807008941 17578603 PMC2870839

[11] Kaufmann A, Kenfak-Foguena A, Andre C, Canellini G, Burgisser P, Moradpour D, Hepatitis E virus seroprevalence among blood donors in southwest Switzerland. PLoS ONE. 2011;6:e21150. 10.1371/journal.pone.0021150 21701586 PMC3118806

[12] Faramawi MF, Johnson E, Chen S, Pannala PR. The incidence of hepatitis E virus infection in the general population of the USA. Epidemiol Infect. 2011;139:1145–50. 10.1017/S0950268810002177 20854712

[13] Mansuy JM, Bendall R, Legrand-Abravanel F, Saune K, Miedouge M, Ellis V, Hepatitis E virus antibodies in blood donors, France. Emerg Infect Dis. 2011;17:2309–12. 10.3201/eid1712.110371 22172156 PMC3311200

[14] Lewis HC, Wichmann O, Duizer E. Transmission routes and risk factors for autochthonous hepatitis E virus infection in Europe: a systematic review. Epidemiol Infect. 2010;138:145–66. 10.1017/S0950268809990847 19804658

[15] Krumbholz A, Mohn U, Lange J, Motz M, Wenzel JJ, Jilg W, Prevalence of hepatitis E virus-specific antibodies in humans with occupational exposure to pigs. Med Microbiol Immunol (Berl). 2012;201:239–44. 10.1007/s00430-011-0210-5 21773797

