# Supplementary material for: Hepatitis E Virus Seroprevalence among Adults, Germany
Source: Emerg Infect Dis. 2012 Oct;18(10):1654–7. doi: 10.3201/eid1810.111756 (PMC3471611; doi:10.3201/eid1810.111756)
Supplement: Technical Appendix — Details of mathematical methods used to compute the yearly incidence from the seroprevalence data in the manuscript. [file 11-1756-Techapp-s1.pdf]

Article DOI: http://dx.doi.org/10.3201/eid1810.111756

Hepatitis E Virus Seroprevalence among Adults, Germany

Technical Appendix

Detailed Methods

Description of the catalytic model

The incidence in the manuscript was computed using a so called simple catalytic epidemic model (Griffiths 1974; Farrington 2005), where the force of infection (FOI) is assumed to be time constant. The dependent variable in the fitted models was a binary variable indicating whether a person in the study had seroconverted or not. Catalytic models assume that infection induces life-long immunity and does not affect the mortality rate of infected individuals. A consequence of the constant FOI is that the population is assumed to be homogeneous with respect to both susceptibility and exposure to infection. Furthermore, infection is assumed to be in equilibrium state, i.e. the level of incidence is assumed to remain constant in time.

Notation

Denote the available data { (y_i,a_i), i = 1,…,n} where y_i is a binary variable indicating if the i'th individual has seroconverted (0 = no, 1 = yes) and a_i is the age of the individual in years (i.e. taken as a continuous variable). Let p(a) be the probability that an individual of age a has sero-converted. Inference about the parameters in a parametric model for p(a) can now be performed using the binomial likelihood:

$$L = \prod_{i=1}^{n} p(a_i)^{y_i} (1 - p(a_i))^{1-y_i}.$$

In the constant FOI model, i.e. $\lambda(a) = \alpha$ for $a \geq 0$, the probability to have seroconverted at age a is given by $p(a) = 1 - \exp(-\alpha a)$. One can show (see, e.g., Becker 1989 or Farrington 2005) that the desired estimation problem for α can be reduced to the fitting of a generalized linear model with complementary log-log (cloglog) link function having the following linear predictor:

$$\eta(a) = \log(-\log(1 - p(a)) = \log(\alpha) + \log(a).$$

This model can be fitted with any GLM software (e.g., function glm in Stata or R) by specifying a binomial model with cloglog link function and using log(a) as offset in the linear predictor. The natural exponent of the intercept estimate in such a model is the desired estimate α. The annual incidence can now be computed as

$$I = \frac{p(a+1) - p(a)}{p(a)} = 1 - \exp(-\alpha).$$

In its basic form, this is the model used by Faramawi et al. (2011) to compute the annual incidence. Confidence intervals for *I* are easily obtained by transforming the confidence interval of the intercept in the GLM by the above equation for *I*. We chose this simple constant FOI in order to obtain a nation-wide estimate of the annual incidence which allows for comparison with the Faramawi et al. estimates for the US.

Results

Performing the above calculations for our n = 4,352 individuals we obtain $\hat{I}$ = 0.00398, i.e., the annual incidence is 398 per 100,000 population with a 95% CI of 372–428. To address post-stratification in the analysis, we additional fitted the above GLM by weighting each observation according to its specific post-strafication sampling weight (e.g. function svyglm in Stata or R). The (survey- weighted) estimated annual incidence is now 392 per 100,000 population (95% CI 364–423). We report this weighted estimate of the annual incidence in the manuscript together with the associated confidence intervals.

Model Checking

Graphical analysis of the residuals from a model with binary response is difficult due to the extreme discrete nature of the problem. Furthermore, such model checking is further complicated by the complex survey setup of our sample. Instead, we perform an alternative examination of the model fit. As a first qualitative assessment, we decided to investigate the models point-wise predictive performance. Based on the asymptotic normality of the GLM estimate we sampled 999 FOI estimates from the normal distribution with mean $\hat{\alpha}$ and variance equal to the estimated variance of $\hat{\alpha}$. For each FOI obtained from this sampling we then

- Calculated the model predicted probability $\hat{p}(a_i)$ for each individual i = 1,…,4352 in the data and sampled a Bernoulli variable using this probability for each individual.
- Given the above Bernoulli realizations we could then for each year of age obtain a new raw seroprevalence estimate respecting the sampling weights (i.e., by using the function svymean).

The Technical Appendix Figure shows point-wise 95% prediction intervals for the seroprevalence of age (in years) based on these 999 parametric bootstrap samples. For comparison, the original survey weighted estimates are indicated as black dots with size proportional to the number of observations available at that age. We observe that the simple catalytic model obtains a good fit with only few points outside the prediction bands. Also, the Pearson goodness of fit test for the simple catalytic model does not reject the null hypothesis (p = 0.88).

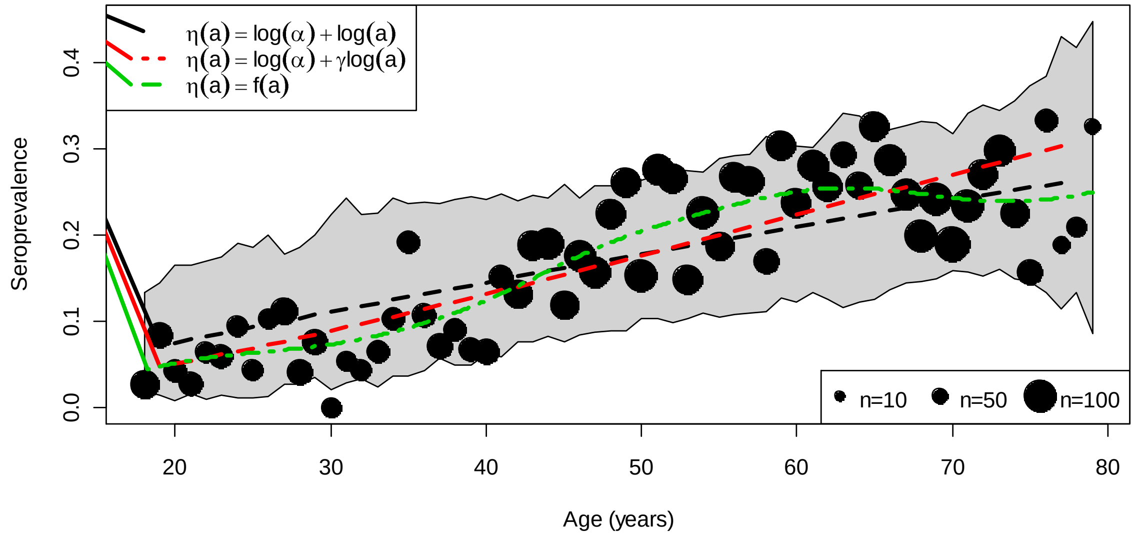

Technical Appendix Figure. Survey-weighted seroprevalence for each year illustrated as black dots. Each dot is shown proportional in size to the actual sample size (n) at that age. 95% point-wise prediction intervals for the simple catalytic are shown in grey. Also shown are the model fitted proportions for the simple catalytic model and two additional models with extra flexibility for age.

In the figure, the black dotted line shows the estimated p(a) of the simple catalytic model. As a sensitivity analysis the red line similarly shows p(a) in Weibull model for the FOI. This model obtains a better fit with γ being significant indicating that the FOI, and hence the annual

incidence, is age specific. However, age specific incidence rates would be harder to interpret and report, especially because our aim was to calculate an overall incidence rate. Another reason is that even this model is not fully sufficient to address all aspects of the data: the green line shows the fit of a fully flexible survey-weighted kernel smoother for p(a). In concordance with Figure 1 of the manuscript we here observe a slight decrease in the seroprevalence at the high ages, which is mentioned in the manuscript discussion. Reporting age-specific annual incidence rate based on such a flexible model would require a table containing a number for each year of age, which is not really useful for our purpose.

Within the cloglog GLM framework it is possible to allow for heterogeneity in the FOI by adjusting for additional variables than age. We investigated additional dependence of sex and residence in the linear predictor, but none of these variables turned out to be significant at the 5% significance level.

Thus, our reported annual incidence estimate remains a nicely interpretable and communicable result which allows for comparison with Faramawi et al., while the above model checking indicates that the assumptions of the constant rate model are reasonable.

References

1. Becker, N. G. (1989). Analysis of Infectious Disease Data, Chapman and Hall.

2. Farrington, P. (2005). Chapter: Communicable diseases. In: Armitage, Peter and Coulton, Theodore eds. Encyclopedia of Biostatistics, 2nd Edition. John Wiley and Sons.

3. Griffiths (1974). A Catalytic Model of Infection for Measles, Journal of the Royal Statistical Society, Series C (Applied Statistics), 23(3):330–339.
